# Bone turnover predicts change in volumetric bone density and bone geometry at the radius in men

**DOI:** 10.1007/s00198-016-3816-z

**Published:** 2016-11-04

**Authors:** S. R. Pye, K. A. Ward, M. J. Cook, M. R. Laurent, E. Gielen, H. Borghs, J. E. Adams, S. Boonen, D. Vanderschueren, F. C. Wu, T. W. O’Neill

**Affiliations:** 1 Arthritis Research UK Centre for Epidemiology, Division of Musculoskeletal and Dermatological Sciences, School of Biological Sciences, Faculty of Biology, Medicine and Health, Manchester Academic Health Science Centre, The University of Manchester, Oxford Road, Manchester, M13 9PT UK; 2 NIHR Manchester Musculoskeletal Biomedical Research Unit, Central Manchester NHS Foundation Trust, Manchester Academic Health Sciences Centre, Manchester, UK; 3 MRC Human Nutrition Research, Elsie Widdowson Laboratory, Cambridge, UK; 4 Gerontology and Geriatrics, Department of Clinical and Experimental Medicine, KU Leuven, Leuven, Belgium; 5 Center for Metabolic Bone Diseases, University Hospitals Leuven, Leuven, Belgium; 6 Radiology and Manchester Academic Health Science Centre, The Royal Infirmary, The University of Manchester, Manchester, UK; 7 Clinical and Experimental Endocrinology, Department of Clinical and Experimental Medicine, KU Leuven, Leuven, Belgium; 8 Andrology Research Unit, Centre for Endocrinology and Diabetes, University of Manchester, Manchester, UK

**Keywords:** Ageing, Bone turnover, Epidemiology, Osteoporosis, Peripheral quantitative computer tomography

## Abstract

**Summary:**

Peripheral quantitative computed tomography scans of the distal and midshaft radius were performed in 514 European men aged 40–79 years at baseline and a median of 4.3 years later. Age-related changes in volumetric bone mineral density (vBMD) and bone geometry were greater in men with higher biochemical markers of bone turnover at baseline.

**Introduction:**

This study aimed to determine prospective change in bone density and geometry at the radius in men and examine the influence of bone turnover markers and sex hormones on that change.

**Methods:**

Men aged 40–79 years were recruited from population registers in Manchester (UK) and Leuven (Belgium). At baseline, markers of bone formation (P1NP and osteocalcin) and resorption (β-cTX and ICTP) were assessed. Total and bioavailable testosterone and oestradiol were also measured. Peripheral quantitative computed tomography (pQCT) was used to scan the radius at distal and midshaft sites at the baseline assessment and a median of 4.3 years later.

**Results:**

Five hundred fourteen men, mean (SD) age of 59.6 (10.5) years, contributed to the data. At the midshaft site, there was a significant decrease in mean cortical vBMD (−0.04 %/year), bone mineral content (BMC) (−0.1 %/year) and cortical thickness (−0.4 %/year), while total and medullary area increased (+0.5 and +2.4 %/year respectively). At the distal radius, total vBMD declined (−0.5 %/year) and radial area increased (+0.6 %/year). Greater plasma concentrations of bone resorption and formation markers were associated with greater decline in BMC and cortical area at the midshaft and total vBMD at the distal site. Increased bone resorption was linked with an increase in total and medullary area and decrease in cortical thickness at the midshaft. Sex hormone levels were unrelated to change in pQCT parameters.

**Conclusions:**

Age-related changes in vBMD and bone geometry are greater in men with higher biochemical markers of bone turnover at baseline. Sex hormones have little influence on change in pQCT parameters.

## Introduction

Osteoporosis in men is a considerable public health problem with the lifetime risk of fracture in men after age 50 years estimated at ~20 % [1]. Most studies examining changes in bone health with age have focussed on ‘areal’ bone mineral density (aBMD; g/cm^2^) [2] as measured by dual-energy X-ray absorptiometry (DXA) [3–8]. However, bone strength is influenced not only by bone mineral content but also by bone shape and mineral distribution and the loading conditions to which the bone is subjected. In addition, DXA tends to overestimate aBMD in larger, and underestimate in smaller, bones [9]. Peripheral quantitative computed tomography (pQCT) allows assessment of both bone geometry and volumetric BMD (vBMD). Data from cross-sectional studies, including data from the European Male Ageing Study (EMAS), suggest variously a lower distal radius vBMD and bone mineral content (BMC), thinner cortices and greater cross-sectional bone area with increasing age [10–17]. However, there are limitations to estimating true longitudinal change in bone parameters from cross-sectional data. In contrast to our understanding about prospective change in DXA aBMD, there are relatively few data concerning prospective change in pQCT parameters in middle-aged and elderly men [18–20], with few data on change in bone geometry at the midshaft and distal radius.

In older men, cross-sectional studies suggest that increased bone turnover markers are associated with lower aBMD [21, 22] and, more recently, microarchitectural parameters [23]. In line with these findings, prospective data suggest that higher levels of bone remodelling may be associated with increased rates of bone loss [24, 25]; however, there are no data linking bone turnover markers to changes in bone geometry in older men.

Levels of sex steroids are known to be associated with aBMD in men, as assessed using DXA, and also rate of bone loss [7, 21, 26–32]. The contribution of oestradiol (E_2_) to BMD has been reasonably well established, but the effect of testosterone (T) is less clear as are the effects of sex hormones on bone structural parameters [33]. Khosla et al. [16] showed that E_2_ was the most constant predictor of BMD and some geometrical variables, assessed by QCT, and similarly in the MINOS cohort, E_2_ was related to aBMD and cortical thickness [26]. Using data from the baseline EMAS survey, we showed a weak association between vBMD and E_2_, while the association of T with bone geometry was inconsistent [17].

The aims of this prospective study were firstly, to characterise longitudinal changes in bone density and structure at the radius in middle-aged and elderly European men; secondly, to determine the relationship between bone turnover markers and subsequent change in BMD and bone structure; and thirdly, to determine the association between sex hormones and change in BMD and structure.

## Materials and methods

### Subjects

The subjects included in this analysis were recruited for participation in the EMAS, a prospective study of ageing in European community-dwelling men. Detailed methods have been described previously [34]. Briefly, men were recruited from population-based sampling frames in eight centres between 2003 and 2005. Stratified random sampling was used with the aim of recruiting equal number of men in each of four 10-year age bands: 40–49, 50–59, 60–69 and 70–79 years. Letters of invitation were sent to subjects asking them to attend for health assessments by a range of health questionnaires, physical performance tests, anthropometry and a fasting blood sample. In two centres, Manchester (UK) and Leuven (Belgium), subjects had pQCT measurements performed at the radius. The men were invited to participate in a follow-up assessment in a median of 4.3 years later. Ethics approval for the study was obtained in accordance with local institutional requirements in each centre, and each participant gave written informed consent.

### Peripheral pQCT

Peripheral QCT measurements of the non-dominant radius were made in men recruited to the Manchester and Leuven centres at both baseline and follow-up using XCT-2000 scanners (Stratec, Pforzheim, Germany). At the distal (4 %) site, total and trabecular vBMD (mg/cm^3^) and bone cross-sectional area (mm^2^) were measured (voxel size 0.4 mm); the slice location at the 4 and 50 % sites was more distal in Leuven compared to Manchester; the reference line was placed at the distal border of the radial end plate in Leuven, and in Manchester, the line was placed to bisect the lateral border of the end plate. These differences resulted in a scan site difference of approximately 1–2 mm between the centres. At the diaphysis (50 % site, voxel size 0.6 mm), cortical vBMD (mg/cm^3^); BMC (mg/mm); total, cortical and medullary areas (mm^2^); cortical thickness (mm); and stress strain index ((SSI) mm^3^) were measured. SSI provides a measure of a bone’s torsional strength [35, 36]. A detailed methodology for these measurements has been described previously [37].

For cross-calibration between Leuven and Manchester, the European forearm phantom (EFP) was measured [38]. There were no differences greater than precision error for trabecular, total and cortical BMD, BMC or cortical area, therefore no cross-calibration was performed between the two centres [17]. The short-term precision of two repeat radius measurements with repositioning in Manchester (*n*=22) and Leuven (*n*=40), respectively were as follows: trabecular BMD 1.27 and 1.42 %; total BMD 2.1 and 1.3 %; cortical BMD 0.77 and 0.71 %; and cortical area 2.4 and 1.3 %. The manufacturer’s standard quality assurance procedures were followed in both centres.

### Bone marker measurement

Bone turnover markers were measured at baseline on the Elecsys 2010 automated analyser (Roche Diagnostics GmbH, Mannheim, Germany). To assess bone resorption, serum beta C-telopeptide of type I collagen (β-cTX) was measured at baseline using the ß-Crosslaps/serum reagents [39]. This assay is specific for cross-linked ß-isomerised type I collagen C-telopeptide fragments and uses two monoclonal antibodies, each recognising the Glu-Lys-Ala-His-ßAsp-Gly-Gly-Arg peptide (Crosslap antigen). The intra-assay coefficient of variation (CV) evaluated by repeated measurements of several serum samples was <5.0 %. The detection limit was 10 pg/mL. Carboxyterminal telopeptide region of type I collagen (ICTP) was measured using the competitive radioimmunoassay technique. A known amount of labelled ICTP and an unknown amount of unlabelled ICTP in the sample compete for the limited number of high-affinity binding sites of the antibody. After separating the free antigen, the amount of labelled ICTP in the sample tube is inversely proportional to the amount of ICTP in the sample. The concentrations in the unknown samples are obtained from a calibration curve. The intra-assay CV was <9 %, and the lower detection limit was <0.4 μg/L. To evaluate bone formation, measurements were performed on the Elecsys 2010 with a two-site assay using monoclonal antibodies raised against intact human P1NP purified from human amniotic fluid. This assay detects both intact monomeric and trimeric forms (total P1NP), as previously described [40]. The intra-assay CV was <3.0 %, and the lower detection limit was <5 ng/mL. The Elecsys N-MID osteocalcin assay uses two monoclonal antibodies specifically directed against epitopes on the N-MID fragment as well as the intact osteocalcin. The test is non-dependent on the unstable C-terminal fragment of the osteocalcin molecule and thus ensures constant measurement results under routine conditions in the laboratory. The intra-assay CV was <4 %, and the lower detection limit was <0.5 ng/mL.

### Sex hormone measurement

A single fasting morning (before 10.00 h) venous blood sample was obtained from all subjects at the baseline assessment. Serum was separated immediately after phlebotomy and stored at −80 °C until assay at the end of the baseline study. Measurement of T and E_2_ was carried out by gas chromatography-mass spectrometry (GC-MS) as described in Labrie et al. [41, 42]. The lower limit of T quantitation was 0.17 nmol/L and of E_2_ was 7.34 pmol/L. The coefficients of variation of T measurements were 2.9 % within runs and 3.4 % between runs and for E_2_ were 3.5 % within runs and 3.7 % between runs. Sex hormone-binding globulin (SHBG) was measured by the Modular E170 platform electrochemiluminescence immunoassay (Roche Diagnostics, Mannheim, Germany) as previously described [43]. Free and bio T and E_2_ levels were derived from total T, total E_2_, SHBG and albumin concentrations using mass action equations and association constants of Vermeulen et al. and Van Pottelbergh et al. [29, 44].

### Statistical analysis

Descriptive statistics were used to summarise subject characteristics at baseline. The change in pQCT parameters was calculated as percentage change per year ((follow-up value − baseline value) / baseline value × 100 / time between scans). Differences between baseline and follow-up pQCT parameters were assessed using paired *t* tests. Linear regression analysis was used to investigate the association of change in pQCT parameters with markers of bone turnover (osteocalcin, P1NP and ICTP and B-cTX) and sex hormones including total and bioavailable E2 and T. In the linear regression analyses, bone turnover markers and sex hormones were standardised (Z-score), so the results represent the change in pQCT parameters per standard deviation increase in the independent variable. Adjustments were made in these analyses for age, height, weight and centre, and the results were expressed as standardised (Z-score) β coefficients and 95 % confidence intervals (CIs). Statistical analysis was performed using Stata version 13 (StataCorp, College Station, TX).

## Results

### Subject characteristics

Five hundred forty men had baseline and follow-up assessments. Of these, 26 were excluded because of therapy which may have impacted on bone including sex hormones, antiosteoporotic therapies and glucocorticoids. Of the 514 included in the analysis, the mean (standard deviation) age was 59.6 (10.5) years and the mean (standard deviation) BMI was 27.3 (3.8) kg/m^2^, see Table 1.

**Table 1:** Subject characteristics

	Mean (SD)
Age at interview (years)	59.6 (10.5)
Height (cm)	174.9 (7.1)
Weight (kg)	83.5 (13.2)
Body mass index (kg/m^2^)	27.3 (3.8)
Testosterone (nmol/L)	18.2 (6.0)
Free testosterone (pmol/L)	320.0 (87.1)
Bioavailable testosterone (nmol/L)	8.0 (2.3)
Oestradiol (pmol/L)	77.8 (24.9)
Free oestradiol (pmol/L)	1.3 (0.4)
Bioavailable oestradiol (pmol/L)	54.6 (17.5)
SHBG (nmol/L)	42.8 (18.4)
P1NP (ng/mL)	42.7 (20.3)
Osteocalcin (ng/mL)	22.3 (7.9)
β-cTX (pg/mL)	327.5 (155.6)
ICTP (ng/mL)	3.1 (0.9)

*SD* standard deviation, *SHBG* sex hormone-binding globulin, *P1NP* serum N-terminal propeptide of type 1 procollagen, *β-cTX* β-C-terminal cross-linked telopeptide, *ICTP* carboxyterminal telopeptide of type I collagen

### Change in bone mass and geometry

There was significant change in most pQCT parameters over the course of the study, see Table 2. At the midshaft radius, mean cortical BMC and vBMD decreased by −0.1 (*P* = 0.03) and −0.04 % (*P* = 0.007) per year respectively, while the medullary and total area increased by 2.4 % (*P* = 0.0001) per year and 0.5 % (*P* = 0.0001) per year respectively. Cortical thickness declined by 0.4 % (*P* < 0.001) per year, with no significant change in cortical area or SSI. At the distal radial site, there was a significant reduction in total vBMD (0.5 % per year, *P* < 0.0001) while radial area increased (0.6 % per year, *P* < 0.0001). In this sample of men age 40–79 years, there was no association between the age and the rate of change of the pQCT parameters (data not shown).

**Table 2:** Radial pQCT parameters: baseline and follow-up

	Mean (SD) value at baseline	Mean (SD) value at follow-up	Mean (SD) % change per year	*P* value^a^
Midshaft radius				
Cortical vBMD (mg/cm^3^)	1214.6 (29.9)	1212.7 (30.1)	−0.04 (0.3)	0.007
Cortical BMC (mg/mm)	124.0 (17.3)	123.5 (17.6)	−0.1 (1.3)	0.03
Total area (mm^2^)	150.3 (21.6)	152.5 (21.1)	0.5 (2.1)	0.0001
Cortical area (mm^2^)	107.5 (13.9)	107.3 (14.1)	−0.06 (1.2)	0.18
Cortical thickness (mm)	3.3 (0.4)	3.2 (0.4)	−0.4 (2.3)	<0.0001
Medullary area (mm^2^)	42.7 (17.0)	45.2 (17.2)	2.4 (6.7)	0.0001
Stress strain index (mm^3^)	342.9 (66.5)	341.5 (64.1)	−0.006 (1.8)	0.3
Distal radius				
Total vBMD (mg/cm^3^)	398.7 (73.2)	391.6 (73.6)	−0.5 (1.4)	<0.0001
Radial area (mm^2^)	381.1 (68.0)	387.8 (69.9)	0.6 (2.5)	<0.0001
Trabecular vBMD (mg/cm^3^)	207.1 (42.3)	206.7 (41.8)	−0.02 (1.4)	0.3

*SD* standard deviation, *BMD* bone mineral density (mg/cm^3^), *BMC* bone mineral content (mg/mm), *vBMD* volumetric bone mineral density

^a^ *P* value for difference between baseline and follow-up value using a paired *t* test

### Influence of bone turnover on change in pQCT parameters

#### Midshaft

After adjustment for age, height, weight and centre, an increase in bone resorption markers (ICTP and β-cTX) as well as bone formation markers (PINP and osteocalcin) were associated with a significant reduction in cortical BMC, see Table 3. An increase in ICTP was associated with a significant increase in both total area (β per SD change = 0.25 % per year) and medullary area (β per SD change = 0.93 % per year). Markers of bone resorption (β-cTX and ICTP) were associated with a greater decline in cortical thickness in the adjusted model. P1NP, osteocalcin and β-cTX were associated with a greater decline in cortical area, see Table 3.

**Table 3:** Influence of bone turnover markers on change in pQCT parameters (% change/year) at the radius

pQCT parameters (% change/year)	P1NP (per (SD)		Osteocalcin (per SD)		β-cTX (per SD)		ICTP (per SD)	
	β coefficient (95 % CI)							
	Unadjusted	Adjusted^a^	Unadjusted	Adjusted^a^	Unadjusted	Adjusted^a^	Unadjusted	Adjusted^a^
Midshaft radius								
Cortical vBMD (mg/cm^3^)	−0.01 (−0.04, 0.01)	−0.01 (−0.04, 0.02)	−0.02 (−0.05, 0.01)	−0.02 (−0.05, 0.01)	−0.02 (−0.04, 0.01)	−0.01 (−0.04, 0.01)	0.00 (−0.03, 0.03)	−0.01 (−0.04, 0.02)
Cortical BMC (mg/mm)	−0.14 (−0.25, −0.03)**	−0.14 (−0.24, −0.03)*	−0.15 (−0.26, −0.04)**	−0.14 (−0.25, −0.03)*	−0.18 (−0.29, −0.07)**	−0.17 (−0.28, −0.06)**	−0.14 (−0.25, −0.03)*	−0.14 (−0.25, −0.02)*
Total area (mm^2^)	0.02 (−0.17, 0.22)	0.04 (−0.16, 0.23)	0.03 (−0.16, 0.22)	0.10 (−0.10, 0.29)	0.12 (−0.07, 0.32)	0.16 (−0.03, 0.36)	0.33 (0.14, 0.52)**	0.25 (0.05, 0.45)*
Cortical area (mm^2^)	−0.13 (−0.23, −0.02)*	−0.12 (−0.22, −0.01)*	−0.13 (−0.24, −0.03)*	−0.11 (−0.22, 0.00)*	−0.16 (−0.26, −0.05)**	−0.14 (−0.24, −0.03)*	−0.09 (−0.19, 0.02)	−0.09 (−0.20, 0.02)
Cortical thickness (mm)	−0.19 (−0.39, 0.01)	−0.19 (−0.38, 0.01)	−0.16 (−0.36, 0.04)	−0.19 (−0.39, 0.01)	−0.27 (−0.47, −0.07)**	−0.27 (−0.47, −0.08)**	−0.38 (−0.58, −0.18)**	−0.33 (−0.53, −0.13)**
Medullary area (mm^2^)	0.16 (−0.44, 0.77)	0.16 (−0.45, 0.78)	0.07 (−0.54, 0.68)	0.18 (−0.46, 0.81)	0.35 (−0.26, 0.96)	0.42 (−0.21, 1.04)	1.00 (0.40, 1.61)**	0.93 (0.29, 1.58)**
Stress strain index (mm^3^)	−0.08 (−0.23, 0.07)	−0.08 (−0.23, 0.07)	−0.10 (−0.25, 0.05)	−0.10 (−0.26, 0.06)	−0.10 (−0.25, 0.06)	−0.09 (−0.25, 0.06)	−0.03 (−0.18, 0.12)	−0.03 (−0.20, 0.13)
Distal radius								
Total vBMD (mg/cm^3^)	−0.16 (−0.29, −0.04)*	−0.16 (−0.29, −0.04)*	−0.07 (−0.20, 0.06)	−0.08 (−0.21, 0.05)	−0.13 (−0.25, 0.00)	−0.14 (−0.27, −0.01)*	−0.06 (−0.19, 0.06)	−0.05 (−0.18, 0.09)
Radial area (mm^2^)	−0.03 (−0.25, 0.19)	−0.03 (−0.26, 0.19)	−0.08 (−0.31, 0.14)	−0.09 (−0.32, 0.15)	−0.09 (−0.31, 0.13)	−0.09 (−0.32, 0.14)	0.00 (−0.22, 0.22)	−0.01 (−0.24, 0.23)
Trabecular vBMD (mg/cm^3^)	−0.08 (−0.20, 0.03)	−0.08 (−0.20, 0.03)	−0.10 (−0.22, 0.01)	−0.09 (−0.21, 0.03)	−0.13 (−0.25, −0.02)*	−0.13 (−0.24, −0.01)*	0.06 (−0.05, 0.17)	0.02 (−0.10, 0.14)

*SD* standard deviation, *P1NP* serum N-terminal propeptide of type 1 procollagen, *β-cTX* β-C-terminal cross-linked telopeptide, *ICTP* carboxyterminal telopeptide of type I collagen, *vBMD* volumetric bone mineral density, *BMC* bone mineral content, *CSMA* cross-sectional muscle area

^a^Change in pQCT parameters per standard deviation increase in bone turnover parameter marker. pQCT parameters are % change per year

^b^Adjusted for age, centre, height and weight

*P < 0.05

**P < 0.01

#### Distal radius

After adjustment for age, height, weight and centre, an increase in β-cTX and P1NP was associated with a reduction in total vBMD (β per SD change = −0.14 and −0.16 % per year respectively). β-cTX was also associated with a reduction in trabecular vBMD (β per SD change = −0.13 % per year).

### Influence of sex hormones on change pQCT parameters

The association between free and bioavailable fractions of T and E_2_ with pQCT parameters was broadly similar, so here we present data for the total and bioavailable hormone relationships (bioE_2_, bioT), see Table 4. There was no association between bio T and E_2_ nor SHBG (data not shown) on change in any of the pQCT parameters in the adjusted models.

**Table 4:** Influence of sex hormones on change in pQCT parameters (% change/year) at the radius

Dependent variable pQCT parameters (% change/year)	Total testosterone (per SD)		Bioavailable testosterone (per SD)		Total oestradiol (per SD)		Bioavailable oestradiol (per SD)	
	Standardised (Z-score) β coefficient (95 % CI)							
	Unadjusted	Adjusted^a^	Unadjusted	Adjusted^a^	Unadjusted	Adjusted^a^	Unadjusted	Adjusted^a^
Midshaft radius								
Cortical vBMD (mg/cm^3^)	0.00 (−0.03, 0.02)	0.00 (−0.03, 0.03)	−0.01 (−0.04, 0.02)	0.01 (−0.03, 0.04)	0.03 (0.00, 0.05)	0.02 (−0.01, 0.05)	0.02 (−0.01, 0.05)	0.02 (−0.01, 0.05)
Cortical BMC (mg/mm)	−0.03 (−0.15, 0.08)	−0.01 (−0.13, 0.12)	0.06 (−0.06, 0.17)	0.04 (−0.10, 0.17)	−0.03 (−0.15, 0.08)	−0.01 (−0.12, 0.11)	0.02 (−0.09, 0.14)	0.03 (−0.09, 0.14)
Total area (mm^2^)	−0.09 (−0.28, 0.10)	−0.12 (−0.33, 0.08)	−0.19 (−0.38, 0.00)	−0.14 (−0.37, 0.08)	−0.09 (−0.28, 0.10)	−0.09 (−0.28, 0.09)	−0.10 (−0.29, 0.08)	−0.07 (−0.27, 0.12)
Cortical area (mm^2^)	−0.06 (−0.17, 0.05)	−0.04 (−0.15, 0.08)	0.03 (−0.08, 0.14)	0.02 (−0.11, 0.15)	−0.06 (−0.16, 0.05)	−0.03 (−0.14, 0.08)	0.01 (−0.10, 0.11)	0.01 (−0.10, 0.12)
Cortical thickness (mm)	0.00 (−0.20, 0.21)	0.08 (−0.14, 0.29)	0.16 (−0.05, 0.36)	0.14 (−0.10, 0.38)	−0.02 (−0.23, 0.18)	0.01 (−0.19, 0.20)	0.05 (−0.15, 0.25)	0.03 (−0.17, 0.24)
Medullary area (mm^2^)	−0.38 (−0.97, 0.22)	−0.49 (−1.15, 0.16)	−0.42 (−1.01, 0.18)	−0.45 (−1.16, 0.27)	−0.05 (−0.64, 0.54)	−0.06 (−0.66, 0.54)	0.01 (−0.58, 0.60)	0.04 (−0.58, 0.65)
Stress strain index (mm^3^)	−0.03 (−0.19, 0.12)	0.00 (−0.17, 0.18)	−0.02 (−0.18, 0.14)	−0.01 (−0.20, 0.18)	−0.03 (−0.19, 0.12)	−0.04 (−0.20, 0.12)	−0.01 (−0.17, 0.14)	−0.04 (−0.20, 0.13)
Distal radius								
Total vBMD (mg/cm^3^)	−0.02 (−0.14, 0.11)	−0.01 (−0.15, 0.13)	0.04 (−0.09, 0.17)	0.04 (−0.12, 0.19)	−0.05 (−0.18, 0.08)	−0.06 (−0.19, 0.07)	−0.03 (−0.16, 0.10)	−0.05 (−0.18, 0.09)
Radial area (mm^2^)	0.02 (−0.19, 0.24)	0.07 (−0.17, 0.31)	0.08 (−0.14, 0.30)	0.09 (−0.18, 0.35)	0.09 (−0.13, 0.31)	0.09 (−0.13, 0.31)	0.12 (−0.10, 0.34)	0.09 (−0.14, 0.32)
Trabecular vBMD (mg/cm^3^)	0.04 (−0.08, 0.17)	0.03 (−0.11, 0.17)	−0.02 (−0.15, 0.11)	0.04 (−0.12, 0.19)	0.07 (−0.05, 0.20)	0.09 (−0.04, 0.21)	0.04 (−0.08, 0.17)	0.09 (−0.04, 0.22)

*SD* standard deviation, *BMD* bone mineral density, *BMC* bone mineral content, *vBMD* volumetric bone mineral density

^a^Change in pQCT parameters per standard deviation increase in bone turnover parameter marker. pQCT parameters are % change per year

^b^Adjusted for age, centre, height and weight

## Discussion

Our data show evidence in middle-aged and elderly men of a longitudinal change in bone mass and geometry at the radial midshaft with a decline in cortical vBMD, BMC and cortical thickness and an increase in medullary and total area. At the distal radius site, there was a decline in the total volumetric BMD and an increase in radial area. A higher rate of bone turnover at baseline (formation and resorption) was associated with a greater reduction in cortical BMC and cortical area at the midshaft and total vBMD at the distal radius. Increased resorption markers were associated with an increase in total and medullary area, a decrease in cortical thickness at the midshaft and a greater rate of decline in trabecular vBMD at the distal radius. In contrast, sex hormones, within the normal range in our community-dwelling sample of men, appeared to have little influence on the change in vBMD and geometry as measured by pQCT.

A number of cross-sectional studies have looked at the influence of age on pQCT parameters in men [10–17]. In a cross-sectional study of 202 men aged 20–99 years and using a high-resolution pQCT, trabecular area/height at the radius increased with age by 28 %, while other parameters decreased with increasing age, including trabecular BMD (−32 %), trabecular thickness (−16 %), cortical area/height (−5 %), cortical BMD ( −15 %) and cortical thickness (−21 %) [11]. In another cross-sectional study using high-resolution pQCT (HR-pQCT) of men aged 20–80 years, compared to younger men (≤35 years), older men (mean age 80 years) had larger total area, thinner trabeculae and lower total and trabecular BMD at the radius [10]. There are, however, limitations in interpreting these data given their cross-sectional design [18]. There are few prospective studies which have looked at change in bone mass and geometry. Data from the Gothenburg Osteoporosis and Obesity Study [45] showed change in radial pQCT parameters in younger men, around the time of accrual of peak bone mass; however, there are limited data in older men (over 60 years of age). In a 7.5 year prospective study, Specker et al. described rates of change at the 4 and 20 % distal radial sites in three distinct populations of 20–66-year-old men [20]. There were increases in bone cross-sectional area, cortical thinning and decreasing bone strength (at older ages) during follow-up. In the InChianti study [18], Lauretani et al., using tibial pQCT data in 345 men (age 21–101 years), reported a decline in BMD and an increase in medullary and total bone area. In a study using HR-pQCT, Shanbhogue et al. reported an increase in trabecular vBMD at the distal radius in men aged 50 years and older over a median follow-up of 3 years, with no significant change in total vBMD or cortical area though the number of men who were studied was relatively small (88) [46].

Given the paucity of prospective data concerning change in pQCT, it is not surprising that there are few data which have looked at the link between bone turnover markers and bone structural change at the distal radius. Using data from the GOOD study, Darelid et al. reported that osteocalcin (OC) was a positive predictor of an increase in aBMD and BMC at the radius between the ages of 19 and 24 years; also, men in the highest quartile of OC at baseline were more likely to gain in radial cross-sectional area and trabecular vBMD than men in the lowest quartile [47]. These findings, particularly in relation to BMD differ from our findings; however, this almost certainly reflects the fact the GOOD study focused on a much younger cohort of men. Our results suggest that increased turnover, and particularly bone resorption, is linked with structural decay and vBMD loss in older men. Such increased bone turnover may be due to a variety of factors including lifestyle, hormonal and metabolic factors (for example GH-IGF, adrenal, sex steroids, PTH, sclerostin and inflammatory status). While there are some similarities to bone loss in women, it is important to recognise there is a sexual dimorphism in patterns of bone ageing. It seems plausible that the reduction in cortical thickness from endosteal bone resorption would impair bone strength if increased strains did not lead to compensatory periosteal expansion to redistribute the bone over a larger cross-sectional area as a mechanism to maintain bone strength. We observed no overall change in stress strain index, suggesting that biomechanical stability persisted despite the loss in cortical thickness. Redistribution of bone is due to periosteal apposition (indicated by an increase in bone area), and our data are in line with previous studies, suggesting that periosteal bone formation in old age may largely be driven in response to endosteal resorption [33]. In any case, the maintenance of bone strength via this mechanism may be one reason why the incidence of wrist fracture in men, in contrast to women, remains low until latter life, though further studies are needed [1, 15].

There is some evidence, at least in mice and rats, that T may increase periosteal apposition (and thereby increase total area), and certainly in adolescents, T increases periosteal growth [33]. Szulc et al. using DXA data suggested an increase in periosteal apposition with age, though not via an action of T [26]. In contrast, Khosla et al. found an inverse association in men with higher levels of T linked with reduced bone area [16]. Our results, however, showed no significant association between either testosterone or oestrogen and change in bone geometry, suggesting that these are not the primary drivers of structural bone decay in community-dwelling men. Evidence from observational and clinical studies support the view that oestrogen is the most important sex steroid in determining bone mass in men [7, 21, 27, 29, 32], with some evidence of a threshold effect, though studies so far are inconclusive [16, 48]. All but eight men in our cohort had total E_2_ >37 pmol/L. Given the low prevalence of clinically significant hypogonadism in EMAS, however, the study may have been underpowered to examine associations between sex steroids and longitudinal pQCT changes.

The strengths of our study were the population sample and the prospective design. There are, however, some limitations which need to be considered when interpreting the results. The response rates for participation in the baseline survey in Manchester and Leuven were 38.8 and 38.6 % respectively [34]. It is possible that those who did not take part may have differed with respect to their pQCT measurements and also bone turnover markers and also sex steroid levels resulting in an overestimation or underestimation with respect to the true population value, and so caution is required in interpreting the absolute levels of these measurements. However, the main findings, in relation to the relationship between bone turnover markers and sex steroid levels and change in pQCT parameters, were based on internal comparisons among responders and so selection factors are unlikely to have influenced the strength of the observed biological relationships.

One of the key factors in designing the study was to ensure standardisation of the study instruments used in the different participating centres. Hormone and bone turnover marker measurements were performed in a central reference laboratory to minimise assay variability, and gold standard mass spectrometry methods were applied. The same pQCT scanner type and model was used in each centre, and after testing scanner differences with the EFP, no cross-calibration was necessary. In our analysis, with 10 bone parameters, it is possible that a number of the significant findings may have been due to chance. The analysis was, however, based on an a priori hypothesis that bone turnover markers and sex hormones may impact on bone, and setting more conservative thresholds for significance may have increased the likelihood of missing true biological associations. Finally, the data were derived from a European Caucasian population and so the results may not necessarily be extrapolated beyond this setting.

In conclusion, our study provides the first longitudinal characterisation of the gradual BMD and bone geometry changes with age at the radius in middle-aged and elderly European men. Increased bone turnover in such men is predictive of bone loss as measured by pQCT. Sex hormones in the normal range, however, appeared to have no influence on the change in pQCT parameters.
